# Comparative sensitivity of the test with tuberculosis recombinant allergen, containing ESAT6-CFP10 protein, and Mantoux test with 2 TU PPD-L in newly diagnosed tuberculosis children and adolescents in Moscow

**DOI:** 10.1371/journal.pone.0208705

**Published:** 2018-12-21

**Authors:** Liudmila Slogotskaya, Elena Bogorodskaya, Diana Ivanova, Tatiana Sevostyanova

**Affiliations:** Clinical Research, Scientific and Clinical Antituberculosis Center of Moscow Government Department of Clinical Research, Scientific and Clinical Antituberculosis Center of Moscow Government Health Department, Moscow, Russian Federation; Fundació Institut d’Investigació en Ciències de la Salut Germans Trias i Pujol, Universitat Autònoma de Barcelona, SPAIN

## Abstract

**Background:**

A group of Russian scientists has developed Diaskintest, which comprises *Mycobacterium tuberculosis*-specific recombinant proteins CFP10-ESAT6, for skin testing (0.2 μg/0.1 ml).

**Study purpose:**

To evaluate the comparative sensitivity of TST with 2 TU PPD-L and a skin test with tuberculous recombinant allergen (Diaskintest) containing the ESAT6-CFP10 protein in children and adolescents with newly diagnosed active tuberculosis during mass screening in the primary medical service in Moscow.

**Materials and methods:**

The trial was a comprehensive retrospective group study of children and adolescents diagnosed in Moscow with active tuberculosis in 2013–2016, aged 0 to 17 years inclusive.

**Results:**

From 441 patients selected for analysis 408 patients had both tests (TST with 2 TU PPD-L and Diaskintest) performed, in 193 patients both tests were given simultaneously, of them 162 patients were BCG-vaccinated. Comparative results of both tests in 408 patients with tuberculosis: at cut-off ≥ 5 mm, both tests has similar sensitivity: Diaskintest 98.3% (95% CI 97.0–99.6%), TST 98.0% (95% CI 96.7–99.4%), at cut-off ≥10 mm, the sensitivity decreases for both tests: Diaskintest 90.0% (95% CI 87.0–93.0%), TST 88.7% (95% CI 85.6–91.9%), but at cut-off ≥ 15 mm, the decrease in sensitivity is statistically significant: for Diaskintest 61.5% (95% CI 56.7–66.3%), and for TST 46.3% (95% CI 41.4–51.3%), p <0.0001.

The results of simultaneous setting of tests on different hands in 193 people (including 162 BCG-vaccinated), do not differ from the results for 408 people.

The correlation between the results of Diaskintest and TST was significant in all groups.

**Conclusion:**

In children and adolescents with active tuberculosis, Diaskintest of 0.2 μg/ml and the Mantoux test with 2 TU PPD-L have high sensitivity (98%) at a cut-off of 5 mm; however, at cut-off ≥ 15 mm sensitivity is significantly reduced, and the decrease is more pronounced in the Mantoux test. The advantage of Diaskintest is that, unlike the Mantoux test, it has high specificity under the conditions of mass BCG vaccination. The test is simple to carry out, and can be used in mass screening.

## Introduction

The diagnosis and treatment of latent tuberculosis infection (LTBI) is one of the strategies recommended by the World Health Organization (WHO) to combat tuberculosis (TB) worldwide and is part of the WHO strategy for tuberculosis control [1–4]. Due to the low specificity of the Mantoux test, the high frequency of false positives due to cross-sensitization with the BCG vaccine strain *(Mycobacterium bovis BCG)*–difficulties arise in interpreting it. [5–6].

An important stage in improving the methods of diagnosis of tuberculosis was the possibility of studying and deciphering the genome of tuberculosis mycobacteria, which allowed to identify the differences between *M*. *bovis BCG* vaccine strain and *Mycobacterium tuberculosis virulent strains*. In particular, in *M*. *tuberculosis* genome, a region of difference *(RDI)* was discovered, which contains genes that code for the secretion of CFP10 and ESAT6 proteins [7–9]. The discovery of antigens specific for *M*. *tuberculosis* led to the development of *in vitro* tests based on the production of gamma-interferon (IFN-γ) in response to stimulation with these antigens (IGRA-Interferon-Gamma Release Assays) [10–11]. The tests showed almost 100% specificity, but a lower sensitivity–about 70–75% [12–15].

The results of IGRA tests in the children's population are mixed, because the confirmation or exclusion of disease of tuberculosis in children is a difficult task [16–20].

The Russia vaccination policy for TB is all children are to be vaccinated with BCG after birth and revaccinated at the age of 7 provided that they have a negative Mantoux test with 2 TU PPD-L.

IGRA tests, having high specificity, still have a number of serious disadvantages: high material costs, the need for an equipped laboratory, intravenous manipulation and the precautionary requirements for maintaining the viability of lymphocytes producing INF-γ, which does not allow this method to be used for mass diagnostics.

The solution of the problem for Russia was the introduction of a skin test with CFP10 and ESAT6 proteins. In Russia, the medicinal product Diaskintest (manufactured by Generium according to the GMP standard) was developed, which is a complex of CFP10/ESAT6 recombinant proteins, produced by *Escherichia coli*, *BL21 (DE3) / pCFP-ESAT*, intended for setting an intradermal test [21]. The test has been widely used in Russia since 2009 according to the order of the Ministry of Health of Russia.

**Study purpose:** study of the comparative sensitivity of Mantoux test with 2 TU PPD-L and a skin test with tuberculous recombinant allergen (Diaskintest) containing the ESAT6–CFP10 protein in newly diagnosed tuberculosis in children and adolescents registered in 2013–2016 in Moscow in a mass application of tests in the primary medical service.

## Materials and methods

### Study designs

**Fig 1:**
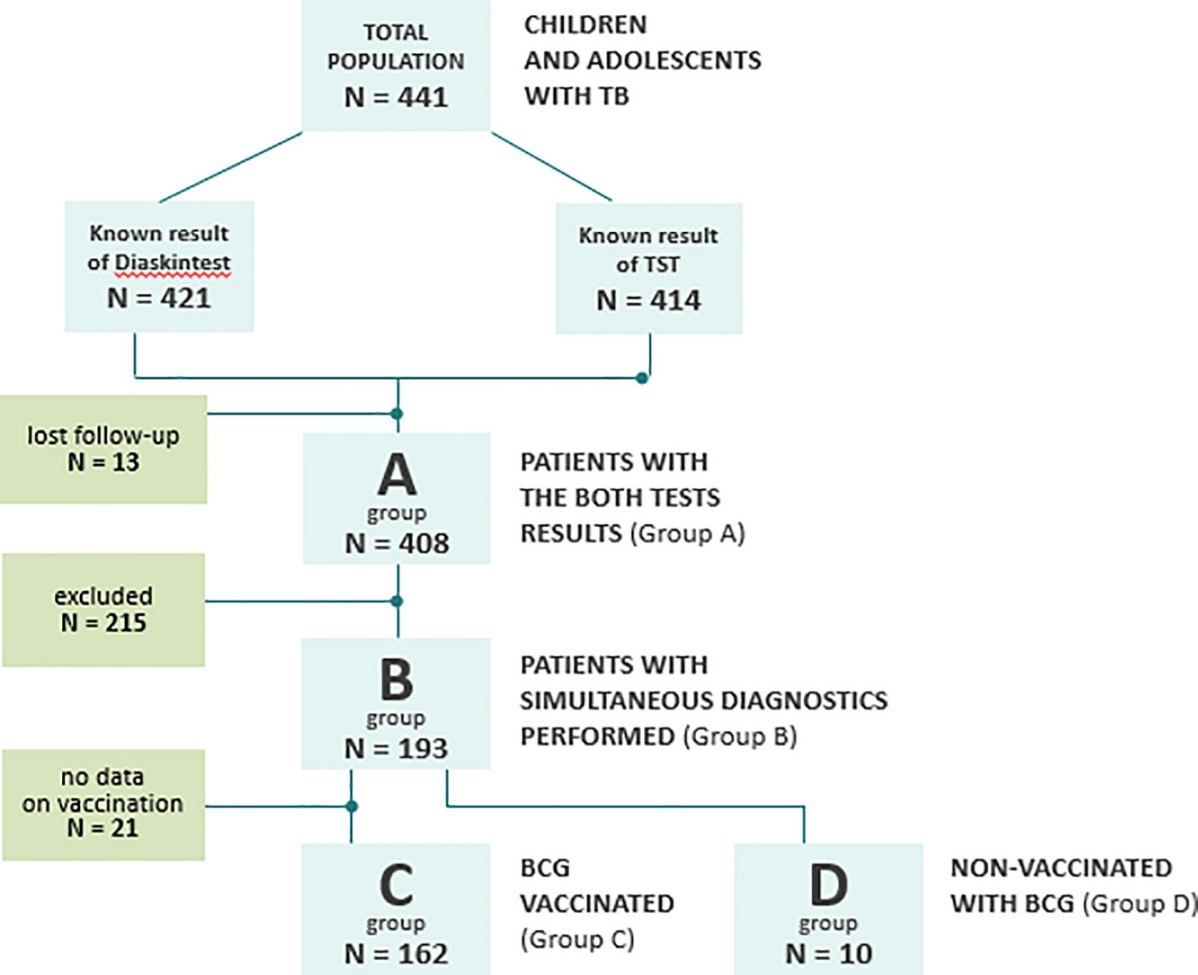
Study flow diagram of screening and testing of tuberculosis infection. TST—tuberculin skin test.

The design of the study–a full-design retrospective group study of children and adolescents diagnosed in Moscow with active tuberculosis in 2013–2016. Inclusion criteria included age at enrolment under of 17 years and the inclusion of active TB disease. There was no formal sample size. The study was conducted as a full-design study in routine practice. The full study schedule is shown in Fig 1.

Every year in Moscow all children and adolescents are screened with the tuberculin skin test with 2TU PPD-L (Saint Petersburg RDE of Vaccines and Serums FMBA of Russia) Children and adolescents with a positive TST (cut-off ≥ 5 mm) should be examined by Diaskintest (CJSC GENERIUM, Russia). Sometimes, at the request of parents, children are given two tests simultaneously on different hands.

Persons with a positive reaction to Diaskintest are subject to a thorough examination to identify active tuberculosis (pulmonary and extrapulmonary). This is a computed tomography of chest organs, ultrasound of other organs, and in the presence of changes in the lungs–a sputum examination on MBT culture and molecular genetic methods. In the presence of local changes in the chest, including the intrathoracic lymph nodes and the pleura, children are subject to a commission assessment of changes in order to establish a diagnosis of tuberculosis or the presence of post-tuberculosis changes. The commission consists of the most qualified phthisiatricians and radiologists. If a diagnosis of tuberculosis is established, the children are hospitalized in a 24–hour in-patient facility and given anti-tuberculosis therapy, depending on the severity of the process, the availability of drug resistance, but at least 6 months. All anti-tuberculosis activities are free, regardless of whether they are permanent Moscow residents or migrants. Children with positive reactions without local changes are subject to preventive therapy for at least 3 months on an outpatient basis or in a sanatorium and are observed during the year.

### Tuberculin skin testing (TST)

TST was performed by the Mantoux method. Two tuberculin units allergen tuberculosis purified (PPD-L, Saint Petersburg RDE of Vaccines and Serums FMBA of Russia) was applied intradermally and transverse induration measured 72 hours later. Positivity was defined as an induration of $5mm and most (cut-off ≥ 5 mm) in accordance with Russian guidelines (stratified threshold).

### Tuberculosis recombinant allergen (Diaskintest)

Tuberculosis recombinant allergen (Diaskintest) (CJSC GENERIUM, Russia) was applied intradermally in a dose of 0.2 μg/0.1 ml according to the Mantoux test technique and transverse induration measured 72 hours later. When evaluating the response to the introduction of the tuberculosis recombinant allergen, the test was regarded as negative in the absence of infiltration or hyperemia, doubtful in the presence of only hyperemia of any size, positive in the presence of an infiltrate of any size.

### Main approaches to the statistical analyses

#### Descriptive statistics

The results contains the descriptive statistics of the subjects included to this study. For quantitative variables (age, the numeric results of the diagnostic tests, etc.) the following statistics presented: number of valid values (N); minimum (Min); maximum (Max); arithmetic mean (M); standard deviation (SD); 95% confidence interval (CI) for the mean; median (Me); interquartile range (IQR). Categorical data (such as gender, a group of the subject, etc.) presented with frequency in a format n/N and percentage (%).

#### Analyses of the test results

Data on sensitivity of the immunological tests presented as frequencies in a format n/N, where n is the number of subjects with true positive test results and N is number of subjects included to the analysis, as well as respective percentage (%).

McNemar test was applied to compare the results on sensitivity and concordance/disconcordance of the two tests and to estimate the odds ratios (OR) for a true positive result under different cut-off levels applied. To reveal chance-corrected agreement between the two tests, the kappa coefficients were calculated. The data presented with all the count a-b-c-d for two-by-two tables under different combinations of the test results.

Quantitative results of the two tests (size of indurations in mm) were analyzed with Pearson correlation and paired t-test. The point estimate for the mean differences in induration sizes along with 95% CI are shown.

#### Ethical approvals

The trial was approved by the Ethics Committee of Scientific and Clinical Antituberculosis Center of Moscow Government Health Department, Moscow (number 3, 17.11.2016) conducted in accordance with the principles of Good Clinical Practice and the World Medical Association (WMA) Declaration of Helsinki adopted by the 18th WMA General Assembly, Helsinki, Finland, 1964 and subsequent amendments.

All parents give written informed consent for carrying out skin tests and any manner of examination and treatment. The patients' parents gave their consent to the processing of the study data provided that no personal information is published. Access to medical documents for the conduct of this study was granted on November 17, 2016.

#### Software

All the statistical analyses were performed using Stata ver. 14 (StataCorp LP, www.stata.com. Stata Statistical Software: Release 14. College Station T:SL, 2).

## Results and discussion

### Results

#### Analyses of baseline characteristics of the subjects

The following age category of subjects was included in the study: children and adolescents aged 0 to 17 years inclusive–total 441. Out of 441 patients, the results of the Diaskintest are known in 421 people, TST- in 414 people (the reasons of no TST results were: parents refusal to perform TST having only Diaskintest done; no data for patients who are not residents, although they were in the territory of Moscow, entered the official statistics of morbidity, received treatment in federal healthcare institutions and left for home, without submitting documents to the Moscow TB Service). The mean age of the patients is 8.8 ± 5.8 years. Data on the average age of subjects in each of these subgroups are summarized below (Table 1). 234 patients (55.6%) were female and 187 (44.4%) were male (S1 Table).

**Table 1:** Descriptive statistics of the patients’ age (years), n = 421.

Statistics	All patients	Patients with both the test results present	Patients with simultaneous diagnostics performed	Vaccinated patients with simultaneous diagnostics performed	Non-vaccinated patients with simultaneous diagnostics performed
N	421	408	193	162	10
M	8.8	8.9	9.5	9.9	4.5
SD	5.7	5.7	5.9	5.6	5.3
95% CI	(8.3; 9.4)	(8.3; 9.4)	(8.7; 10.4)	(9.0; 10.8)	(7.9; 9.4)
Min	0.1	0.1	0.1	0.4	0.5
Max	17.9	17.9	17.9	17.8	16.9
Me	7.8	7.9	9.3	10.2	2.6
IQR	11.4	11.3	11.4	11.1	4.8

N—number of valid values, M—arithmetic mean; SD—standard deviation; 95% CI—95% confidence interval (CI) for the mean; Min–minimum; Max–maximum; Me–median; IQR—interquartile range.

Out of 421 patients, 408 patients had the results of both tests, 193 patients were simultaneously given 2 tests: TST with 2 TU PPD-L and Diaskintest, 162 of whom were BCG-vaccinated, 10 were not vaccinated, no data were reported for 21, due they were migrants.

The diagnosis of tuberculosis is confirmed by the discovery of the pathogen in a small number of cases (13/421–3.1%). The main diagnosis is based on a combination of clinical and radiological data (S3 Table).

#### Analyses of the sensitivity of the two tests

Of 421 patients having results of Diaskintest, the negative reaction was found within 8/421 (1.9%) subjects, while 9/421 (2.1%) had induration size of less than 5 mm. Thus, the sensitivity of Diaskintest at cut-off > 0 mm was 98.1% (95% CI 96.2%-99.2%), at cut-off ≥ 5 mm 97.9% (95% CI 96.0%-99.0%, at cut-off ≥ 10 mm– 89.8% (95% CI 86.5%-92.5%, and at cut-off ≥ 15 mm 61,1% (95% CI 56.2%-65.7%) (Table 2).

**Table 2:** Sensitivity of the tests on the sample of all the patients, n = 421.

Diagnostic test	сut-off > 0 mm	сut-off ≥ 5 mm	сut-off ≥ 10 mm	сut-off ≥ 15 mm
Diaskintest (n = 421) 95% CI	413/421 (98.1%) 96.2%-99.2%	412/421 (97.9%) 96.0%-99.0%	378/421 (89.8%) 86.5%-92.5%	257/421 (61.1%) 56.2%-65.7%
TST (n = 414) 95% CI	409/414 (98.8%) 97.2%-99.6%	406/414 (98.1%) 96.2%-99.2%	368/414 (88.9%) 85.5%-91.7%	191/414 (46.1%) 41.3%-51.1%

Diaskintest—skin test with tuberculous recombinant allergen; TST—tuberculin skin test; cut-off–size of induration; 95% CI—95% confidence interval (CI) for the mean

TST results were available for 414 patients (the parents of the rest have refused to perform TST having done only the Diaskintest). Absence of induration has been revealed for 5/414 (1.2%) subjects, while 8/414 (1.9%) had induration of less than 5 mm. Thus, the sensitivity of TST was 98.8% (95% CI 97.2%-99.6%), 98.1% (95% CI 96.2%-99.2%), 88.9% (95% CI 85.5%-91.7%), and 46.1% (95% CI 41.3%-51.1%), respectively (Table 2). Sensitivity at cut-off **≥** 15 mm was statistically significant lower compared to that of Diaskintest (р<0.0001).

**Fig 2:**
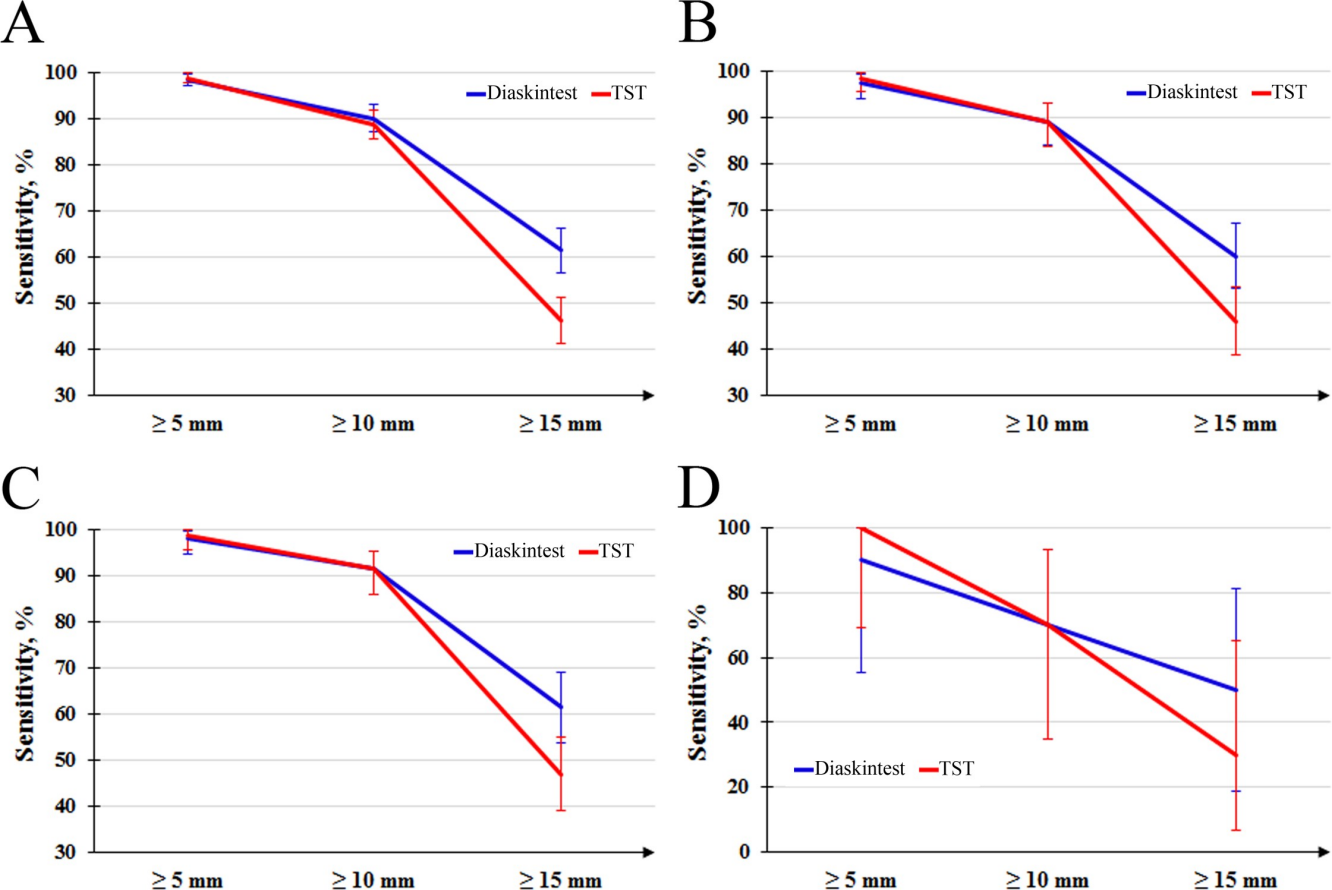
Sensitivity of the two diagnostics tests (a point estimation along with 95% CI): A—the patients with the both results present (n = 408), B—patients with simultaneous diagnostics performed (n = 193), C—vaccinated patients with simultaneous diagnostics performed (n = 162), D—non-vaccinated patients with simultaneous diagnostics performed (n = 10).

408 of 421 subjects had the results of both the tests (Table 3). It was found that at cut-off ≥ 5 mm the sensitivity of both the tests was similar: Diaskintest 98.3% (95% CI 97.0–99.6%) and TST 98.0% (95% CI 96.7–99.4%), while at cut-off ≥ 10 mm the sensitivity somewhat decrease to 90.0% (95% CI 87.0–93.0%) for Diaskintest and to 88.7% (95% CI 85.6–91.9%) for TST. However, at cut-off ≥15 mm the drop in sensitivity was both statistically and clinically significant (р< 0.0001): to 61.5% (95% CI 56.7–66.3%) for Diaskintest and even worse for TST–to 46.3% (95% CI 41.4–51.3%) (Fig 2).

**Table 3:** Sensitivity of the tests among the patients having both the tests results, n = 408.

Diagnostic test	0 mm	сut-off > 0 mm	сut-off ≥ 5 mm	сut-off ≥ 10 mm	сut-off ≥ 15 mm
Diaskintest (n = 408) 95% CI	6/408 (1.5%) 0.3%-2.7%	402/408 (98.5%) 97.3%-99.7%	401/408 (98.3%) 97.0%-99.6%	367/408 (90.0%) 87.0%-93.0%	251/408 (61.5%) 56.7%-66.3%
TST (n = 408) 95% CI	5/408 (1.2%) 0.1%-2.3%	403/408 (98.8%) 97.7%-99.9%	400/408 (98.0%) 96.7%-99.4%	362/408 (88.7%) 85.6%-91.9%	189/408 (46.3%) 41.4%-51.3%

Diaskintest—skin test with tuberculous recombinant allergen; TST—tuberculin skin test; cut-off–size of induration; 95% CI—95% confidence interval (CI) for the mean

The simultaneous diagnostics with both the tests was performed for 193 subjects (Table 4, Fig 2).

**Table 4:** Sensitivity of the tests among the patients with simultaneous diagnostics performed, n = 193.

Diagnostic test	0 mm	сut-off > 0 mm	сut-off ≥ 5 mm	сut-off ≥ 10 mm	сut-off ≥ 15 mm
Diaskintest 95% CI	5/193 (2.6%) 0.8%-5.9%	188/193 (97.4%) 94.1%-99.2%	188/193 (97.4%) 94.1%-99.2%	172/193 (89.1%) 83.9%-93.2%	116/193 (60.1%) 53.1%-67.2%
TST 95% CI	2/193 (1.0%) 0.1%-3.7%	191/193 (99.0%) 96.3%-99.9%	190/193 (98.5%) 95.5%-99.7%	172/193 (89.1%) 83.8%-93.1%	89/193 (46.1%) 38.9%-53.4%

Diaskintest—skin test with tuberculous recombinant allergen; TST—tuberculin skin test; cut-off–size of induration; 95% CI—95% confidence interval (CI) for the mean

Among patients with the simultaneous using of the two different skin tests, 162 persons (94.2%) were known to be BCG vaccinated (Table 5, Fig 2). Sensitivity of both the tests was similar to that of the sample collected 193 patients, as almost all of them were vaccinated.

**Table 5:** Sensitivity of the tests among the vaccinated patients with simultaneous diagnostics performed, n = 162.

Diagnostic test	сut-off > 0 mm	сut-off ≥ 5 mm	сut-off ≥ 10 mm	сut-off ≥ 15 mm
Diaskintest 95% CI	159/162 (98.2%) 94.7%-99.6%	159/162 (98.2%) 94.7%-99.6%	148/162 (91.4%) 85.9%-95.2%	100/162 (61.7%) 53.8%-69.2%
TST 95% CI	161/162 (99.4%) 96.6%-100.0%	160/162 (98.8%) 95.6%-99.9%	148/162 (91.4%) 85.9%-95.2%	76/162 (46.9%) 39.0%-54.9%

Diaskintest—skin test with tuberculous recombinant allergen; TST—tuberculin skin test; cut-off–size of induration; 95% CI—95% confidence interval (CI) for the mean

As BCG vaccination is mandatory in the newborn and revaccination–in children at the age of 7 years (with negative Mantoux reactions with 2 TU PPD-L), the number of non-vaccinated patients was only 10 subjects, which explains the extremely wide confidence intervals observed.

The comparison of the results between TST and Diaskintest on the sample of patients with the both diagnostic tests present was performed using McNemar test at different cut-off levels (Table 6). Given the high concordance of the test results, the kappa coefficient, adjusted for the probability of random coincidence of the results was not so high (kappa <0.4), while no statistically significant difference between the two test results was revealed when an equal cut-off level (either ≥ 5 mm or ≥ 10 mm) was applied for both the tests (p > 0.05). It is also interesting to note, that at cut-off ≥ 15 mm Diaskintest was 2.7 times more likely (p < 0.0001) to show a true positive result (OR = 0.37 with 95% CI 0.25–0.55).

**Table 6:** Comparison of the results between TST and Diaskintest at different cut-off levels on the sample of patients with the both results present, n = 408.

TST		Diaskintest							
		>0 mm		≥ 5 mm		≥ 10 mm		≥ 15 mm	
Cut-off	Result	Neg.	Pos.	Neg.	Pos.	Neg.	Pos.	Neg.	Pos.
**≥ 5 mm**	Neg.	1 (0.3%)	7 (1.7%)	1 (0.3%)	7 (1.7%)	4 (1.0%)	4 (1.0%)	7 (1.7%)	1 (0.3%)
	Pos.	5 (1.2%)	395 (96.8%)	6 (1.5%)	394 (96.6%)	37 (9.1%)	363 (89.0%)	150 (36.8%)	250 (61.3%)
	Agreement, %	97.1		96.4		90.0		63.0	
	P (McNemar) OR (95% CI)	0.774 0.71 (0.18; 2.61)		1.000 0.86 (0.24; 2.98)		<0.0001 9.25 (3.32; 35.73)		<0.0001 150.00 (26.51; 5963.68)	
**≥ 10 mm**	Neg.	4 (1.0%)	42 (10.3%)	4 (1.0%)	42 (10.3%)	19 (4.7%)	27 (6.6%)	33 (8.1%)	13 (3.2%)
	Pos.	2 (0.5%)	360 (88.2%)	3 (0.7%)	359 (88.0%)	22 (5.4%)	340 (83.3%)	124 (30.4%)	238 (58.3%)
	Agreement, %	87.6		89.0		88.0		66.4	
	P (McNemar) OR (95% CI)	<0.0001 0.05 (0.01; 0.18)		<0.0001 0.07 (0.01; 0.22)		0.568 0.81 (0.44; 1.49)		<0.0001 9.54 (5.38; 18.42)	
**≥ 15 mm**	Neg.	5 (1.2%)	214 (52.5%)	5 (1.2%)	214 (52.5%)	34 (8.3%)	185 (45.3%)	120 (29.4%)	99 (24.3%)
	Pos.	1 (0.3%)	188 (46.1%)	2 (0.5%)	187 (45.8%)	7 (1.7%)	182 (44.6%)	37 (9.1%)	152 (37.3%)
	Agreement, %	47.3		47.1		52.9		66.7	
	P (McNemar) OR (95% CI)	<0.0001 0.00 (0.00; 0.03)		<0.0001 0.01 (0.00; 0.03)		<0.0001 0.04 (0.02; 0.08)		<0.0001 0.37 (0.25; 0.55)	

Diaskintest—skin test with tuberculous recombinant allergen; TST—tuberculin skin test; cut-off–size of induration; 95% CI—95% confidence interval (CI) for the mean; Neg.—negative result test; Pos.–positive result test; OR—the odds ratios

Similar results were obtained for the test of patients with simultaneous diagnostic tests performed (Table 7). No statistically significant difference between the two test results was revealed when an equal cut-off level (either ≥ 5 mm or ≥ 10 mm) was applied for both the tests (p > 0.05). It is also interesting to note, that at cut-off ≥ 15 mm Diaskintest was 2.9 times more likely (p = 0.0004) to show a positive results (OR = 0.34 with 95% CI 0.17–0.64). In subjects with simultaneous tests with TST cut-off ≥ 10 mm and Diaskintest cut-off ≥ 0 mm, the chances of obtaining a positive result of Diaskintest were at least 2.17 times higher (OR = 0.11 with 95% CI 0.01–0.46).

**Table 7:** Comparison of the results between TST and Diaskintest at different cut-off levels on the sample of patients with simultaneous diagnostics performed, n = 193.

TST		Diaskintest							
		>0 mm		≥ 5 mm		≥ 10 mm		≥ 15 mm	
Cut-off	Result	Neg.	Pos.	Neg.	Pos.	Neg.	Pos.	Neg.	Pos.
**≥ 5 mm**	Neg.	1 (0.5%)	2 (1.0%)	1 (0.5%)	2 (1.0%)	2 (1.0%)	1 (0.5%)	3 (1.6%)	0 (0.0%)
	Pos.	4 (2.1%)	186 (96.4%)	4 (2.1%)	186 (96.4%)	19 (9.8%)	171 (88.6%)	74 (38.3%)	116 (60.1%)
	Agreement, %	96.9		96.9		89.6		61.7	
	P (McNemar) OR (95% CI)	0.688 2.00 (0.29; 22.11)		0.688 2.00 (0.29; 22.11)		<0.0001 19.00 (3.02; 789.46)		<0.0001 - (19.56; -)	
**≥ 10 mm**	Neg.	3 (1.6%)	18 (9.3%)	3 (1.6%)	18 (9.3%)	11 (5.7%)	10 (5.2%)	17 (8.8%)	4 (2.1%)
	Pos.	2 (1.0%)	170 (88.1%)	2 (1.0%)	170 (88.1%)	10 (5.2%)	162 (83.9%)	60 (31.1%)	112 (58.0%)
	Agreement, %	89.7		89.7		89.6		66.8	
	P (McNemar) OR (95% CI)	0.0004 0.11 (0.01; 0.46)		0.0004 0.11 (0.01; 0.46)		1.000 1.00 (0.37; 2.68)		<0.0001 15.00 (5.56; 56.84)	
**≥ 15 mm**	Neg.	4 (2.1%)	100 (51.8%)	4 (2.1%)	100 (51.8%)	19 (9.8%)	85 (44.0%)	63 (32.6%)	41 (21.2%)
	Pos.	1 (0.5%)	88 (45.6%)	1 (0.5%)	88 (45.6%)	2 (1.0%)	87 (45.1%)	14 (7.3%)	75 (38.9%)
	Agreement, %	47.7		47.7		54.9		71.5	
	P (McNemar) OR (95% CI)	<0.0001 0.01 (0.00; 0.06)		<0.0001 0.01 (0.00; 0.06)		<0.0001 0.02 (0.00; 0.09)		0.0004 0.34 (0.17; 0.64)	

Diaskintest—skin test with tuberculous recombinant allergen; TST—tuberculin skin test; cut-off–size of induration; 95% CI—95% confidence interval (CI) for the mean; Neg.—negative result test; Pos.–positive result test; OR—the odds ratios

The results obtained for the BCG vaccinated patients with simultaneous diagnostics performed (Table 8) were also similar. While no statistically significant difference between the two test results was revealed when an equal cut-off level (either ≥ 5 mm or ≥ 10 mm) was applied for both the tests (p > 0.05). It is also interesting to note, that at cut-off ≥ 15 mm Diaskintest was 2.8 times more likely (p = 0.0009) to show a true positive result (OR = 13/37 = 0.35 with 95% CI 0.17–0.68).

**Table 8:** Comparison of the results between TST and Diaskintest at different cut-off levels on the sample of vaccinated patients with simultaneous diagnostics performed, n = 162.

TST		Diaskintest							
		>0 mm		≥ 5 mm		≥ 10 mm		≥ 15 mm	
Cut-off	Result	Neg.	Pos.	Neg.	Pos.	Neg.	Pos.	Neg.	Pos.
**≥ 5 mm**	Neg.	0 (0.0%)	2 (1.2%)	0 (0.0%)	2 (1.2%)	1 (0.6%)	1 (0.6%)	2 (1.2%)	0 (0.0%)
	Pos.	3 (1.9%)	157 (96.9%)	3 (1.9%)	157 (96.9%)	13 (8.0%)	147 (90.7%)	60 (37.0%)	100 (61.7%)
	Agreement, %	96.9		96.9		91.3		62.9	
	P (McNemar) OR (95% CI)	1.000 1.50 (0.17; 17.96)		1.000 1.50 (0.17; 17.96)		0.0018 13.00 (1.95; 552.47)		<0.0001 - (15.77; -)	
**≥ 10 mm**	Neg.	1 (0.6%)	13 (8.0%)	1 (0.6%)	13 (8.0%)	5 (3.1%)	9 (5.6%)	10 (6.2%)	4 (2.5%)
	Pos.	2 (1.2%)	146 (90.1%)	2 (1.2%)	146 (90.1%)	9 (5.6%)	139 (85.8%)	52 (32.1%)	96 (59.3%)
	Agreement, %	90.7		90.7		88.9		65.5	
	P (McNemar) OR (95% CI)	0.0074 0.15 (0.02; 0.68)		0.0074 0.15 (0.02; 0.68)		1.000 1.00 (0.35; 2.84)		<0.0001 13.00 (4.78; 49.50)	
**≥ 15 mm**	Neg.	2 (1.2%)	84 (51.9%)	2 (1.2%)	84 (51.9%)	12 (7.4%)	74 (45.7%)	49 (30.3%)	37 (22.8%)
	Pos.	1 (0.6%)	75 (46.3%)	1 (0.6%)	75 (46.3%)	2 (1.2%)	74 (45.7%)	13 (8.0%)	63 (38.9%)
	Agreement, %	47.5		47.5		53.1		69.2	
	P (McNemar) OR (95% CI)	<0.0001 0.01 (0.00; 0.07)		<0.0001 0.01 (0.00; 0.07)		<0.0001 0.03 (0.00; 0.10)		0.0009 0.35 (0.17; 0.68)	

Diaskintest—skin test with tuberculous recombinant allergen; TST—tuberculin skin test; cut-off–size of induration; 95% CI—95% confidence interval (CI) for the mean; Neg.—negative result test; Pos.–positive result test; OR—the odds ratios

The results for non-vaccinated patients with simultaneous tests were not statistically significant due to a small sample size (n = 10) (S2 Table).

### Analyses of the size of indurations

**Fig 3:**
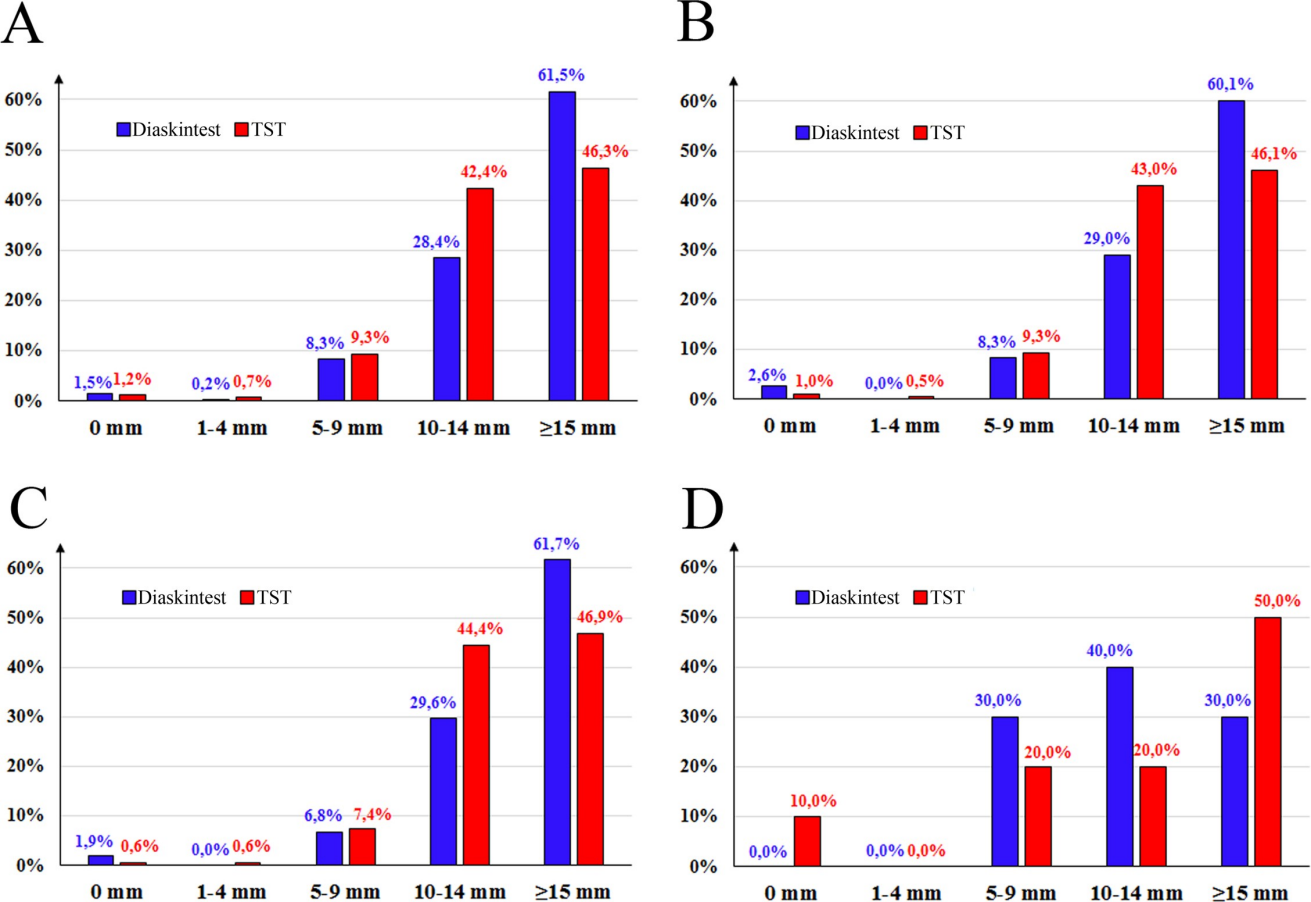
Induration size for the two diagnostics tests: A—the patients with the both results present (n = 408), B—patients with simultaneous diagnostics performed (n = 193), C—vaccinated patients with simultaneous diagnostics performed (n = 162), D—non-vaccinated patients with simultaneous diagnostics performed (n = 10).

Most of the patients had the size of induration ≥10 mm for both the diagnostic tests. At the same time, the percent of patients having induration ≥15 mm on Diaskintest test was higher compared to TST (Fig 3): 61.5% vs 46.3% (for all the patients with the both results present, n = 408), 60.1% vs 46.1% (for the patients with simultaneous diagnostics performed, n = 193), 61.7% vs 46.9% (for the vaccinated patients with simultaneous diagnostics performed, n = 162), and 30.0% vs 50.0% (for the non-vaccinated patients with simultaneous diagnostics performed, n = 10).

**Fig 4:**
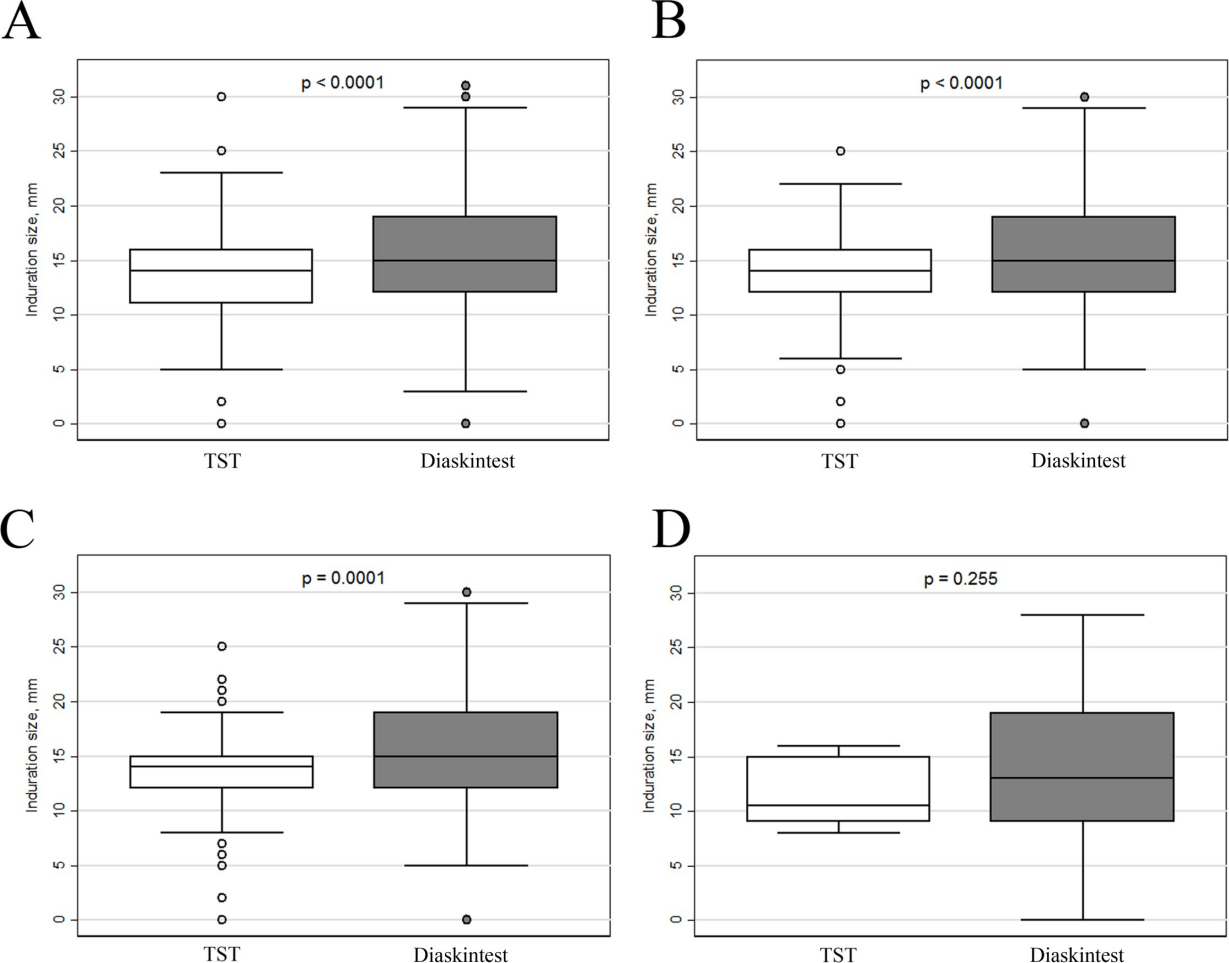
Boxplot for the induration sizes of the two diagnostics tests: A—the patients with the both results present (n = 408), B—patients with simultaneous diagnostics performed (n = 193), C—vaccinated patients with simultaneous diagnostics performed (n = 162), D—non-vaccinated patients with simultaneous diagnostics performed (n = 10).

Generally, the patients in all the samples have shown a higher induration size for Diaskintest compared with TST. The analysis performed with a paired t-test has shown statistically significant differences (p < 0.001) except for the non-vaccinated patients with simultaneous diagnostics performed, where only a small sample size was available (Fig 4, S4 Table). The difference was on average 1.9 mm (with 95% CI 1.4–2.4 mm) for all the patients with both the test results present. Similar effect size was found for the patients with simultaneous diagnostics performed: on average 1.4 mm (with 95% CI 0.8–2.1 mm). Despite for the non-vaccinated patients the difference has not reached a level of statistical significance, the point estimate was also 2.0 mm (13.6 ± 7.8 mm for Diaskintest versus 11.6 ± 3.2 mm for TST).

**Fig 5:**
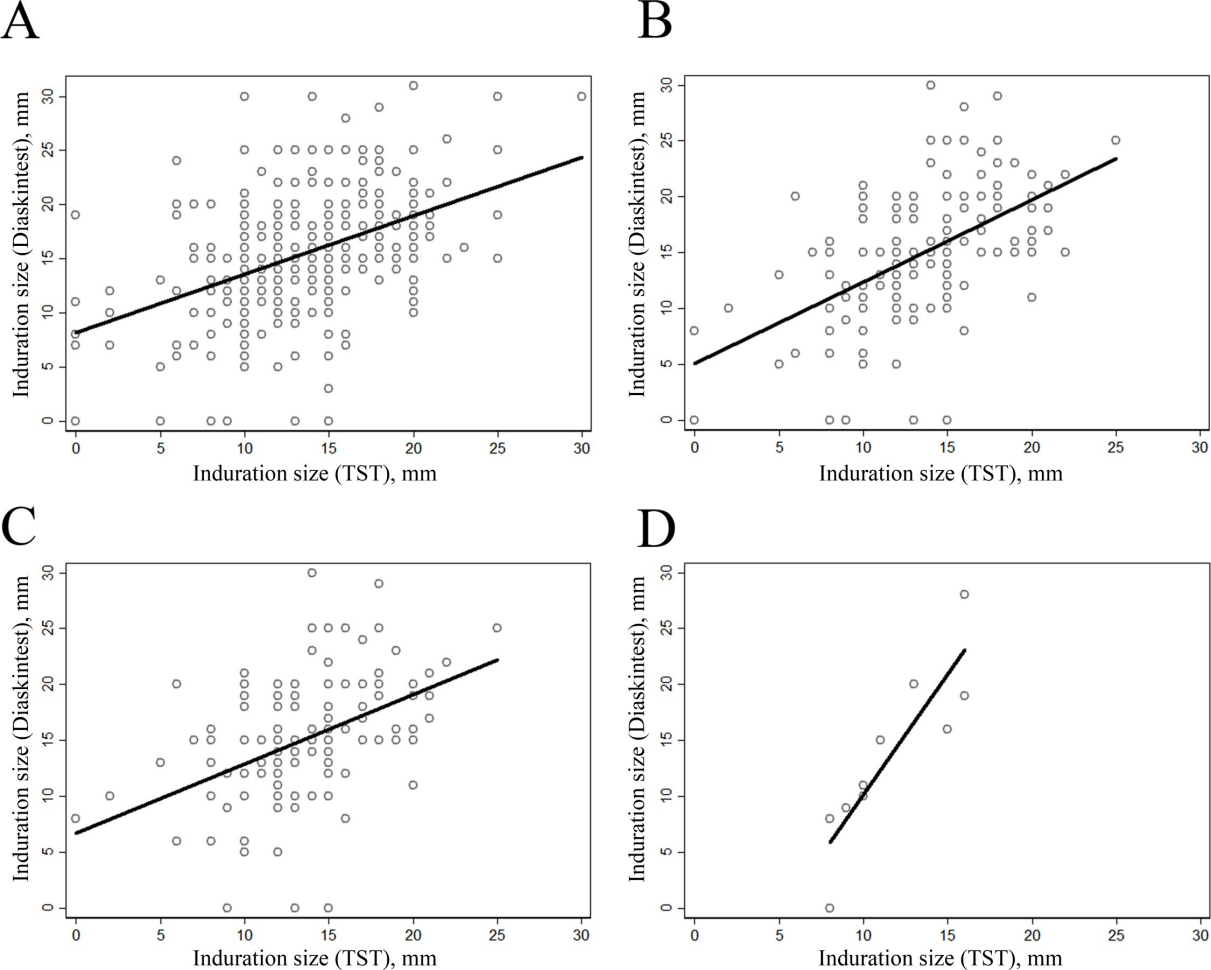
Scatter-plot for the induration sizes of the two diagnostics tests: A—the patients with the both results present (n = 408), B—patients with simultaneous diagnostics performed (n = 193), C—vaccinated patients with simultaneous diagnostics performed (n = 162), D—non-vaccinated patients with simultaneous diagnostics performed (n = 10).

The Pearson correlation between induration sizes of the two tests was of clinical and statistical significance (above 0.4, p < 0.001) in all the samples with maximum of 0.878 observed for the non-vaccinated patients with simultaneous diagnostics performed (Fig 5).

The comparative study of the sensitivity of TST and Diaskintest in the same of 408 children with newly diagnosed tuberculosis of respiratory organs in different cut-offs demonstrated the highest sensitivity of TST and Diaskintest for cut-off ≥ 5 mm: Diaskintest 98.3% (95% CI 97.0–99.6%), TST 98.0% (95% CI 96.7–99.4%), with cut-off ≥10 mm, the sensitivity of tests decreases to Diaskintest insignificantly to TST to a greater extent: 90.0% (95% CI 87.0–93.0%), and 88.7% (95% CI 85.6–91.9%), respectively; but with cut-off ≥ 15 mm, the decrease in sensitivity is statistically significant: 61.5% (95% CI 56.7–66.3%) to Diaskintest, and to TST 46.3% (95% CI 41.4–51.3%), p <0.0001.

Discordant results with negative Diaskintest were observed: in 2013, in 2 children aged 2 and 3 months who were born by women with tuberculosis. Children were examined as contacts–both tests were negative. Another child suffered from chronic adrenal insufficiency (TST (+), Diaskintest (-)), he was examined as contact with a tuberculous father and had disseminated pulmonary tuberculosis; another child with disseminated pulmonary tuberculosis and one with focal pulmonary tuberculosis (Mantoux test (+), Diaskintest (-)). In 2014, one teenager with pleurisy (Mantoux test (+), Diaskintest (-)), in 2015, 4 children with HIV infection and disseminated pulmonary tuberculosis (Mantoux test (-), Diaskintest (-)); 2 teenagers with infiltrative pulmonary tuberculosis (Mantoux test (+), Diaskintest (-)), 1 child with hilar tuberculosis in the calcification phase (Mantoux test (+), Diaskintest (-)), in 2016, 1 child, 6 months old, not vaccinated with BCG, with disseminated tuberculosis had a negative reaction (Mantoux test (+), Diaskintest (-)).

## Discussion

The comparative study of the sensitivity of TST and Diaskintest in the same of 408 children with newly diagnosed tuberculosis of respiratory organs in different cut-offs demonstrated the highest sensitivity of TST and Diaskintest for cut-off ≥ 5 mm: 98%, with cut-off ≥10 mm, the sensitivity of tests decreases to Diaskintest insignificantly to TST to a greater extent: 90.0% and 88.7% respectively; but with cut-off ≥ 15 mm, the decrease in sensitivity is statistically significant: 61.5% to Diaskintest, and to TST 46.3%, p <0.0001(Table 3, Fig 2).

Given the high concordance of the test results, the kappa coefficient, was not so high (kappa <0.4), while no statistically significant difference between the two test results was revealed when an equal cut-off level (either ≥ 5 mm or ≥ 10 mm) was applied for both the tests (p > 0.05). It is also interesting to note, that at cut-off ≥ 15 mm Diaskintest was 2.7 times more likely (p < 0.0001) to show a true positive result (OR = 0.37 with 95% CI 0.25–0.55). (Table 6).

Similar results were obtained for the test of 193 patients with simultaneous diagnostics performed (Tables 4, Fig 2). The results obtained for the BCG vaccinated 162 patients with simultaneous diagnostics performed were also similar (Tables 5, Fig 2).

Most of the patients had the size of induration ≥10 mm for both the diagnostic tests. At the same time, the percent of patients having induration ≥15 mm on Diaskintest test was higher compared to TST (Figs 3 and 4): 61.5% vs 46.3% (for all the patients with the both results present, n = 408).

Recent reviews of WHO strategy END TB specify the priority tasks: development of *biomarkers* for the detection and diagnosis of tuberculosis in children including systematic *screening* (active search for TB cases); detection of *latent tuberculosis infection*–to reduce the pool of individuals with LTBI. A biomarker shall have low cost–for TB diagnostics at the primary healthcare level [22,23]. The review of National policies on the management of latent tuberculosis infection of 98 countries identified that tuberculin skin testing was the most frequently recommended diagnostic tool. The test is comparably cheaper than interferon-gamma release assay. Several countries specified additional diagnostic algorithms, such as different tuberculin skin test cut-off points among specific risk groups, sequential use of the two tests, or use of interferon assay for BCG-vaccinated individuals [24]. The diversity of policies across countries calls for more research in how to use interferon-gamma release assay and tuberculin skin testing together among different risk groups based on the underlying tuberculosis epidemiology [24–26]

The major advantage of skin test with ESAT6 CFP10 in endemic countries would be substantially low cost (will be competitive with TST) [15].

Since there is no diagnostic standard for LTBI, active tuberculosis is used as a comparable substitute, although tuberculosis is known to be immunosuppressive.

In Russia, BCG vaccination of newborns and revaccination at 7 years (with negative TST with 2 TU PPD-L) is mandatory. Mantoux tuberculin test with 2 TU PPD-L was also mandatory so far. It is clear that under such conditions the specificity of the sample is extremely low. The situation is aggravated by the fact that in Russia there is one limit of positive results– 5 mm for all–vaccinated and unvaccinated. At the same time, positive reactions to the Mantoux test in children and adolescents are registered in 75%, and the increase in the reaction is observed annually only in 0.9% of the tuberculin-positive ones. Annually, the rate of primarily infected children among the tuberculin-positive is 0.67%, and 0.06% in adolescents [27]. In such conditions, there was a need for a specific test capable of distinguishing post-vaccination allergy. Such a test was a skin test with a tubercular recombinant allergen (Diaskintest) containing ESAT-6–CFP-10 protein. [21]. Since 2009, at the order of the Ministry of Health of Russia, the Diaskintest has been used for differential diagnosis of postvaccinal and infectious allergies. All children with a positive reaction to Diaskintest should be examined to identify local forms of tuberculosis and, in the absence of such, to receive preventive treatment for LTBI. Detection of tuberculosis among people with positive reactions to Diaskintest is ten times higher than among tuberculin-positive [28].

There is no international agreement on cut-off values for the definition of a positive tuberculin reaction [29–32]. The choice among commonly used cut-off values (e.g. a diameter of induration of ≥ 5mm, ≥ 10mm or ≥ 15mm) depends on an individual’s risk factor profile for TB. Usually, a lower cut-off value of ≥ 5mm is used for individuals at higher risk of TB and a higher cut-off value of ≥ 10mm is applied for individuals at lower risk of TB [30–32].

Similar to the Russian test, the Danish skin test with C-Tb was developed, which is produced by *Statens Serum Institut* (Copenhagen, Denmark)–the difference is that it is a mixture of two recombinant proteins ESAT-6 and CFP-10 in the ratio of 1:1. Both proteins are produced by *Lactococcus lactis*, the dose of the drug for intradermal application is 0.1 μg/0.1 ml [14,15,33,34,35].

In phase III clinical studies of C-tb Danish skin test, three tests were compared: QuantiFERON-TB Gold In-Tube, tuberculin skin test (TST), and C-tb skin test. The results showed that sensitivity of C-Tb was 69% in *TB*, which is 14% lower than that of QFT [14].

We compared the sensitivity results obtained with Diaskintest and the results of the skin test with C-tb [14] and let us assume the reasons for the lower sensitivity of the latter: firstly, adults with lower sensitivity compared to children were examined, and secondly, those who completed the treatment a long time ago were enrolled to the group of those surveyed, and they probably had a negative reaction, as was observed in our studies with Diaskintest [36]; the possibility of insufficient sensitivity in the difference in dosage in C-tb 0.1 μg (Diaskintest– 0.2 μg) is not excluded.

The high sensitivity of Diaskintest in children can be explained by the optimal diagnosis of the disease, which is facilitated by the fact that children from 1 year of age are subject to annual tuberculin diagnostics, the moment of primary infection is fixed and they are subject to examination at the phthisiatrician. Our comparative studies of the sensitivity of Diaskintest and QuantiFERON-TB Gold In-Tube tests showed high sensitivity of not only Diaskintest, but also QuantiFERON-TB Gold In-Tube (more than 90%) [37], which can be explained by the adequate diagnosis of tuberculosis in children, which is a difficult task in the absence of clinical manifestations and bacterial excretion. After the introduction of Diaskintest to wide practice in 2010 and the prescription of preventive therapy to children with a positive reaction, the incidence of tuberculosis in Moscow in 2016 decreased from 10.6 to 5.5. The incidence in adolescents showed a decrease from 28.7 to 11.6, with the incidence of the resident population almost 2 times lower in both children and adolescents [38]. An important role is played by the fact that the detection, diagnosis and treatment of tuberculosis and LTBI in children is fully funded, regardless of the social belonging of children, their citizenship and their permanent place of residence. All undergo the vaccination, annual tuberculin diagnostics, Diaskintest (with positive reactions to TST), computed tomography with positive Diaskintest reaction, bacteriological and molecular genetic examination, long-term controlled therapy in a hospital with tuberculosis, and outpatient or in a sanatorium with LTBI (at the request of parents). Data on the high sensitivity of the skin test with the drug containing the specific ESAT6–CFP10 protein, corresponds to the data of S. Sollai [39] that in high-income countries the sensitivity of IGRA tests is higher than in low-income countries.

Diaskintest test is simple to perform, it is currently used in primary health care, it is cheap (the cost is similar to the Mantoux test) and is now implemented by the order of the Ministry of Health into the practice of Russia as a screening method instead of Mantoux test in children over 7 years old.

## Conclusion

In children and adolescents with tuberculosis, Diaskintest at a dose of 0.2 μg/ml and the Mantoux test with 2 TU PPD-L have a high sensitivity (98%) with a cut-off of 5 mm; however, at cut-off ≥ 15 mm sensitivity is significantly reduced, and the decrease is more pronounced in the Mantoux test. At cut-off ≥ 15 mm Diaskintest was 2.7 times more likely (p < 0.0001) to show a true positive result (OR = 0.37 with 95% CI 0.25–0.55). The advantage of Diaskintest is that, unlike the Mantoux test, it has high specificity under the conditions of mass BCG vaccination. The test is simple to carry out, and can be used in mass screening.

## Supporting information

S1 Table Gender distribution of the patients, n = 421. (DOCX)

S2 Table Comparison of the results between TST and Diaskintest at different cut-off levels on the sample of non-vaccinated patients with simultaneous diagnostics performed (n = 10). (DOCX)

S3 Table Distribution of the patients of the type of tuberculosis, n = 421. (DOCX)

S4 Table Descriptive statistics of the induration size (mm) of the Diaskintest (n = 421) and TST, n = 414. (DOCX)
