# Supplementary material for: Comparative sensitivity of the test with tuberculosis recombinant allergen, containing ESAT6-CFP10 protein, and Mantoux test with 2 TU PPD-L in newly diagnosed tuberculosis children and adolescents in Moscow
Source: PLoS One. 2018 Dec 21;13(12):e0208705. doi: 10.1371/journal.pone.0208705 (PMC6303070; doi:10.1371/journal.pone.0208705)
Supplement: S1 Table — (DOCX) [file pone.0208705.s001.docx]

S1 Table

Gender distribution of the patients, n= 421

| **Gender** | **All patients** | **Patients with both the test results present** | **Patients with simultaneous diagnostics performed** | **Vaccinated patients with simultaneous diagnostics performed** | **Non-vaccinated patients with simultaneous diagnostics performed** |
| --- | --- | --- | --- | --- | --- |
| Females | 234/421 (55.6%) | 229/408 (56.1%) | 113/193 (58.5%) | 93/162 (57.4%) | 6/10 (60.0%) |
| Males | 187/421 (44.4%) | 179/408 (43.9%) | 80/193 (41.5%) | 69/162 (42.6%) | 4/10 (40.0%) |
