# Supplementary material for: Comparative sensitivity of the test with tuberculosis recombinant allergen, containing ESAT6-CFP10 protein, and Mantoux test with 2 TU PPD-L in newly diagnosed tuberculosis children and adolescents in Moscow
Source: PLoS One. 2018 Dec 21;13(12):e0208705. doi: 10.1371/journal.pone.0208705 (PMC6303070; doi:10.1371/journal.pone.0208705)
Supplement: S2 Table — (DOCX) [file pone.0208705.s002.docx]

S2 Table

Comparison of the results between TST and Diaskintest at different cut-off levels on the sample of non-vaccinated patients with simultaneous diagnostics performed (n = 10)

| **TST** | | **Diaskintest** | | | | | | | |
| --- | --- | --- | --- | --- | --- | --- | --- | --- | --- |
|  |  | **>0 mm** | | **≥ 5 mm** | | **≥ 10 mm** | | **≥ 15 mm** | |
| **Cut-off** | **Result** | Neg. | Pos. | Neg. | Pos. | Neg. | Pos. | Neg. | Pos. |
| **≥ 5 mm** | Neg. | 0 (0.0%) | 0 (0.0%) | 0 (0.0%) | 0 (0.0%) | 0 (0.0%) | 0 (0.0%) | 0 (0.0%) | 0 (0.0%) |
|  | Pos. | 1 (10.0%) | 9 (90.0%) | 1 (10.0%) | 9 (90.0%) | 3 (30.0%) | 7 (70.0%) | 5 (50.0%) | 5 (50.0%) |
|  | Agreement, % | 90.0 | | 90.0 | | 70.0 | | 50.0 | |
|  | P (McNemar)  Kappa  OR (95% CI) | 1.000  0.000  - (0.03; -) | | 1.000  0.000  - (0.03; -) | | 0.250  0.000  - (0.41; -) | | 0.063  0.000  - (0.92; -) | |
| **≥ 10 mm** | Neg. | 1 (10.0%) | 2 (20.0%) | 1 (10.0%) | 2 (20.0%) | 3 (30.0%) | 0 (0.0%) | 3 (30.0%) | 0 (0.0%) |
|  | Pos. | 0 (0.0%) | 7 (70.0%) | 0 (0.0%) | 7 (70.0%) | 0 (0.0%) | 7 (70.0%) | 2 (20.0%) | 5 (50.0%) |
|  | Agreement, % | 80.0 | | 80.0 | | 100.0 | | 80.0 | |
|  | P (McNemar)  Kappa  OR (95% CI) | 0.500  0.412  0.00 (0.00; 5.32) | | 0.500  0.412  0.00 (0.00; 5.32) | | 1.000  1.000  - (-; -) | | 0.500  0.600  - (0.19; -) | |
| **≥ 15 mm** | Neg. | 1 (10.0%) | 6 (60.0%) | 1 (10.0%) | 6 (60.0%) | 3 (30.0%) | 4 (40.0%) | 5 (50.0%) | 2 (20.0%) |
|  | Pos. | 0 (0.0%) | 3 (30.0%) | 0 (0.0%) | 3 (30.0%) | 0 (0.0%) | 3 (30.0%) | 0 (0.0%) | 3 (30.0%) |
|  | Agreement, % | 40.0 | | 40.0 | | 60.0 | | 80.0 | |
|  | P (McNemar)  Kappa  OR (95% CI) | 0.0313  0.091  0.00 (0.00; 0.85) | | 0.0313  0.091  0.00 (0.00; 0.85) | | 0.125  0.310  0.00 (0.00; 1.51) | | 0.500  0.600  0.00 (0.00; 5.32) | |

Diaskintest - skin test with tuberculous recombinant allergen; TST - tuberculin skin test; cut-off – size of induration; 95% CI - 95% confidence interval (CI) for the mean; Neg. - negative result test; Pos. – positive result test; OR - the odds ratios
