# Supplementary material for: Comparative sensitivity of the test with tuberculosis recombinant allergen, containing ESAT6-CFP10 protein, and Mantoux test with 2 TU PPD-L in newly diagnosed tuberculosis children and adolescents in Moscow
Source: PLoS One. 2018 Dec 21;13(12):e0208705. doi: 10.1371/journal.pone.0208705 (PMC6303070; doi:10.1371/journal.pone.0208705)
Supplement: S3 Table — (DOCX) [file pone.0208705.s003.docx]

S3 Table

Distribution of the patients of the type of tuberculosis, n=421.

| **Type of tuberculosis** | **All patients** | **Patients with both the test results present** | **Patients with simultaneous diagnostics performed** | **Vaccinated patients with simultaneous diagnostics performed** | **Non-vaccinated patients with simultaneous diagnostics performed** |
| --- | --- | --- | --- | --- | --- |
| Intrathoracic lymph nodes tuberculosis | 200/421 (47.5 %) | 196/408 (48.0 %) | 85/193 (44.0 %) | 71/162 (43.8 %) | 6/10 (60.0 %) |
| Primary tuberculosis complex | 84/421 (20.0 %) | 79/408 (19.4 %) | 33/193 (17.1 %) | 28/162 (17.3 %) | 2/10 (20.0 %) |
| Infiltrative lung tuberculosis | 63/421 (15.0 %) | 62/408 (15.2 %) | 28/193 (14.5 %) | 25/162 (15.4 %) | 1/10 (10.0 %) |
| Focal tuberculosis | 55/421 (13.1 %) | 54/408 (13.2 %) | 36/193 (18.7 %) | 30/162 (18.5 %) | 0/10 (0.0 %) |
| Disseminated tuberculosis | 10/421 (1.9 %) | 9/408 (1.7 %) | 7/193 (2.5 %) | 6/162 (3.1 %) | 1/10 (0.0 %) |
| Tuberculoma | 4/421 (1.0 %) | 4/408 (1.0 %) | 0/193 (0.0 %) | 0/162 (0.0 %) | 0/10 (0.0 %) |
| Cavernous tuberculosis | 2/421 (0.5 %) | 2/408 (0.5 %) | 2/193 (1.0 %) | 0/162 (0.0 %) | 0/10 (0.0 %) |
| Pleurisy of tuberculosis etiology | 3/421 (0.7 %) | 2/408 (0.5 %) | 2/193 (1.0 %) | 2/162 (1.2 %) | 0/10 (0.0 %) |
