# Supplementary material for: Comparative sensitivity of the test with tuberculosis recombinant allergen, containing ESAT6-CFP10 protein, and Mantoux test with 2 TU PPD-L in newly diagnosed tuberculosis children and adolescents in Moscow
Source: PLoS One. 2018 Dec 21;13(12):e0208705. doi: 10.1371/journal.pone.0208705 (PMC6303070; doi:10.1371/journal.pone.0208705)
Supplement: S4 Table — (DOCX) [file pone.0208705.s004.docx]

S4 Table

Descriptive statistics of the induration size (mm) of the Diaskintest (n=421) and TST, n=414.

| **Test** | **Statistics** | **All patients** | **Patients with both the test results present** | **Patients with simultaneous diagnostics performed** | **Vaccinated patients with simultaneous diagnostics performed** | **Non-vaccinated patients with simultaneous diagnostics performed** |
| --- | --- | --- | --- | --- | --- | --- |
| Diaskintest | N | 421 | 408 | 193 | 162 | 10 |
|  | M | 15.4 | 15.5 | 15.1 | 15.3 | 13.6 |
|  | SD | 5.3 | 5.2 | 5.3 | 5.1 | 7.8 |
|  | 95% CI | (14.9; 15.9) | (15.0; 16.0) | (14.4; 15.9) | (14.5; 16.1) | (8.0; 19.2) |
|  | Min | 0 | 0 | 0 | 0 | 0 |
|  | Max | 31 | 31 | 30 | 30 | 28 |
|  | Me | 15 | 15 | 15 | 15 | 13 |
|  | IQR | 7 | 7 | 7 | 7 | 10 |
| TST | N | 414 | 408 | 193 | 162 | 10 |
|  | M | 13.6 | 13.6 | 13.7 | 13.8 | 11.6 |
|  | SD | 4.2 | 4.2 | 3.9 | 3.7 | 3.2 |
|  | 95% CI | (13.2; 14.0) | (13.2; 14.0) | (13.1; 14.2) | (13.3; 14.4) | (9.3; 13.9) |
|  | Min | 0 | 0 | 0 | 0 | 8 |
|  | Max | 30 | 30 | 25 | 25 | 16 |
|  | Me | 14 | 14 | 14 | 14 | 10.5 |
|  | IQR | 5 | 5 | 4 | 3 | 6 |
| P (paired t-test) | | **<0.0001** | **<0.0001** | **<0.0001** | **0.0001** | 0.255 |
| Pearson correlation | | 0.440 | 0.440 | 0.539 | 0.454 | 0.878 |
| Difference (95% CI) | | 1.9 (1.4; 2.4) | 1.9 (1.4; 2.4) | 1.4 (0.8; 2.1) | 1.5 (0.7; 2.2) | 2.0 (-1.7; 5.7) |

N - number of valid values; M - arithmetic mean; SD - standard deviation; 95% CI - 95% confidence interval (CI) for the mean; Min – minimum; Max – maximum; Me – median; IQR - interquartile range, Diaskintest - skin test with tuberculous recombinant allergen; TST - tuberculin skin test; 95% CI - 95% confidence interval (CI) for the mean
